# Beyond the Surface: The Peculiar World of Benthic Biodiversity, from Microbes to Multicellular Life and Their Ecosystem Roles

**DOI:** 10.3390/biology14040368

**Published:** 2025-04-03

**Authors:** Mayya Gogina, Judith Piontek

**Affiliations:** Leibniz Institute for Baltic Sea Research Warnemünde, Seestrasse 15, D-18119 Rostock, Germany; judith.piontek@io-warnemuende.de

Below the water column depths, marine sediments harbor a vibrant tapestry of life that underpins a variety of ecological balances. From the tiniest microbes to complex multicellular organisms, benthic communities play pivotal roles in nutrient cycling, energy flow, and habitat formation. Understanding these intricate ecosystems is paramount, especially as they face intensive evolutionary fluctuation and natural succession. Changing climate conditions and the increasing influence of other threats from human activities are putting pressure on communities in benthic marine ecosystems and require adaptations within short periods of time.

In this Special Issue, we wanted to delve into the multifaceted world of benthic biodiversity, exploring the dynamic interactions among its diverse communities and their impacts on marine ecosystem functioning. We were lucky to collect 13 articles, totaling 252 pages, that have been co-authored by 65 researchers from 8 countries.

The contributions span a broad spectrum of topics, beginning with microbial communities that form the foundation of benthic ecosystems. Advanced metabarcoding techniques have unveiled the astonishing diversity and versatility of benthic protists, shedding light on their roles in biogeochemical processes and food webs and addressing their resilience to environmental perturbations. Transitioning to meiofauna and macrofauna, several studies investigate species distribution patterns in response to natural gradients and anthropogenic pressures like bottom trawling, sea level rise, or organic and pollutant influences from the river plume, suggesting species that suffer most from these perturbations and spotting those that are tolerant. These findings underscore the sensitivity of benthic organisms to habitat alterations, emphasizing the need for comprehensive monitoring and conservation strategies.

Interdisciplinary approaches featured in this issue highlight the functional interplays between different benthic compartments, but also the need for more integrated assessments. For instance, research on bioturbation and bioirrigation demonstrates how macrofaunal activities enhance microbial processes, facilitating nutrient exchange across the sediment–water interface. Such interactions are crucial for maintaining ecosystem health and productivity. However, establishing robust quantitative relationships to improve predictive capacity remains a challenge.

The contributions in this issue primarily focus on observational and modeling approaches to understand benthic biodiversity distribution and ecosystem functioning (Table 1). Purely experimental studies were unfortunately lacking. We therefore advocate for more of them in the future, as they can reveal not only species-specific responses but also potential cascading effects on community structure and ecosystem services. Such insights remain missing for many organism groups but are vital for predicting future changes and formulating adaptive management plans.

To serve as a quick reference for readers to navigate the diverse topics covered in this Special Issue and to synthesize the wealth of information presented, Table 1 categorizes the 13 published studies based on their focal organism groups (microbes, meiofauna, macrofauna, fish) and methodological approaches (observational, experimental, modeling) and outlines key findings related to ecosystem functions. In conclusion, this compilation of research offers a snapshot of some current advancements in benthic biodiversity studies. It underscores the importance of integrative approaches to unravel the complexities of marine ecosystems and informs strategies for their understanding and preservation amidst a rapidly changing world.

Additionally, Figure 1 illustrates schematically the expected interconnections between different benthic biodiversity components, depicting how microbial, meiofaunal, and macrofaunal interactions might drive or alter essential processes like nutrient cycling, carbon remineralization, deoxygenation, and overall energy transfer.

## Figures and Tables

**Figure 1:**
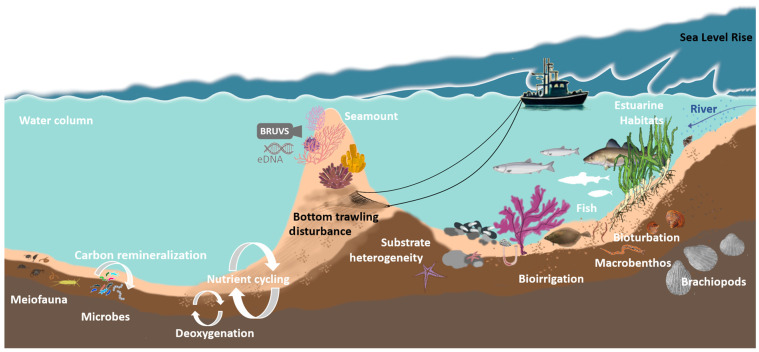
Schematic illustration of the expected interconnections between different benthic biodiversity components and processes (labeled in white), exemplary pressures (labeled in black), as well as some currently actively developed monitoring methods (shown in gray).

**Table 1:** Summary of 13 papers included in this Special Issue, distinguishing between organism groups studied and methodologies and highlighting key findings.

Reference	Focal Organism Group	Methodological Approach	Key Ecosystem Function Finding
[1]	Macrofauna	Observational, Modeling, Experimental	The Bioirrigation Potential index (BIPc) derived from observational macrofauna data correlates with irrigation rates, confirming that macrofaunal activity significantly enhances the efficiency of solute transfer in benthic environments.
[2]	Macrofauna	Observational, Experimental	Bottom trawling shifts benthic communities toward opportunistic, deposit-feeding species that do not bioturbate. This disturbance does not affect oxygen consumption (site- and season-dependent) but reduces carbon mineralization due to the removal of reactive surface sediment. The decline in community complexity and bioturbation leads to decreased sediment oxygenation, a reduction in carbon mineralization, and higher organic carbon concentrations in the sediment.
[3]	Macrofauna	Observational	Sea level rise will modify the seafloor macrofauna communities in estuaries and subsequently alter the related ecosystem functions.
[4]	Macrofauna	Observational	With increasing depth and decreasing influence from the river plume, species density and related sediment mixing and bioirrigation decreased. Highest macrofauna diversity was observed in the upper first cm of sediment, but the highest biomass was in deeper (6 cm) depth layers.
[5]	Macrofauna	Observational	Using microsatellite markers, the genetic diversity of commercially important clams was shown to be influenced by breeding mode and revealed no genetic isolation of populations by distance.
[6]	Macrofauna, brachiopods	Observational, paleo	Based on precise age constraints from conodont biostratigraphy and quantitative brachiopod data, a previous underestimate of the diversity of Olenekian brachiopod fauna diversity is suggested, and brachiopod recovery in the studied section is attributed to the latest Spathian period.
[7]	Macrofauna	Observational	Field assessments document a direct link between macrofaunal diversity and sediment structure. Small patches of different soft sediment types are associated with elevated species richness and a higher rate of occurrence of rare species.
[8]	Macrofauna	Observational	Research on marine bivalves suggested that lipid matrix membranes of the mitochondria of long-lived species are less sensitive to in vitro-initiated peroxidation compared to species with shorter life spans.
[9]	Microbes, protists	Observational	Metabarcoding-based assessment of benthic protists, which act as controllers of bacterial and microphytobenthos production and contribute significantly to the carbon flux, suggested community response to salinity, sediment properties, and oxygen.
[10]	Fish (demersal and benthopelagic), megafauna	Observational	Field surveys using environmental DNA metabarcoding (eDNA) and baited video (BRUVS) revealed no diversity hotspots for fish at seamounts. Shallower seamounts, as biomass oases and refuges for threatened megafauna, were spotted as protection priorities.
[11]	Macrofauna	Observational, Modeling	Field geological and biological surveys and modeling were used to map the distribution of seabed habitats and biotopes and their inhabitants.
[12]	Macrofauna	Observational	Field surveys compare recent benthic macrofauna biodiversity in German Marine Protected Areas of the Baltic and the North Sea along environmental gradients and decreasing bottom trawling intensity.
[13]	Macrofauna	Observational	Seagrass (regardless of density) positively affects macrozoobenthic communities and their functioning, indicating meadows as key biotopes that can support biogeochemical processes in coastal zones more effectively than bare sands.
